# P03-13 Assessing the distribution of adolescents physical activity in each social time

**DOI:** 10.1093/eurpub/ckac095.049

**Published:** 2022-08-29

**Authors:** Thibaut Derigny, Christophe Sschnitzler, Joseph Gandrieau, François Potdevin

**Affiliations:** 1 STAPS, University of Lille, Lille, France; 2 STAPS, University of Strasbourg, Strasbourg, France

**Keywords:** Health, Adolescent, Environment, Ecosystem

## Abstract

Regular physical activity (PA) in youth has mental and social health outcomes (Ekelund, 2016; Biddle, 2019). Available data also suggest that the level of PA in youth predicts PA in adulthood (Telama, 2014; Varma, 2017). However, international studies (Aubert et al, 2018) have shown that the majority of young people do not achieve the PA levels recommended for health benefits (Guthold, 2020). However, despite policy intentions to develop sports infrastructure (Deguilhem, 2016; Esteban, 2016), a decrease in the overall level of physical inactivity will not be achieved by 2025 (Guthold, 2020). It seems to be a gap between the willingness of public services and the concrete actions of citizens. One of the commonly recognized obstacles to PA is the lack of time (Embersin, 2007). Thus, based on Elias' temporal model (1997) we propose to move beyond the linear view of time (chronos) to a perspective of timely time (kairos). Adopting an ecological perspective on human development, postulating that multiple determinants impact on our choices of physical activity (Bauman, 2012), we qualify and quantify the social times conducive to physical activity and inactivity. Using objective measurements of PA by accelerometry (ActiGraph GT3X) and a measure of social time by digital daily diary, we identify three profiles of adolescents whose perceptions of time vary according to their level of PA. Adolescents with an active profile (>3000 METs per week) practice PA within the framework of school and out-of-school by optimizing active transports, whereas those with a low activity profile (600>PA>3000 METs per week) are mainly involved in school PA. Finally, adolescents with an inactive profile (PA > 600 METs per week) only practice during compulsory physical education lessons and no social time records intense PA. As the only social time conducive to PA captured by all adolescents, the physical education lesson does not seem to be able, in its current form, to reduce inequalities in access to daily PA opportunities. There is scope for research into the organization of a school ecology (Waters, 2013; Turcotte, 2018) conducive to health education through PA.

